# Antioxidant Capacity, Phenolic Compounds, and Other Constituents of Cold and Hot Yerba Mate (*Ilex paraguariensis*) Infusions

**DOI:** 10.3390/antiox13121467

**Published:** 2024-11-29

**Authors:** Julia Płatkiewicz, Daniel Okołowicz, Robert Frankowski, Tomasz Grześkowiak, Magdalena Jeszka-Skowron, Agnieszka Zgoła-Grześkowiak

**Affiliations:** Institute of Chemistry and Technical Electrochemistry, Poznan University of Technology, Berdychowo 4, 60-965 Poznań, Poland; julia.platkiewicz@doctorate.put.poznan.pl (J.P.); okolowicz.daniel@gmail.com (D.O.); robert.frankowski@put.poznan.pl (R.F.); civ@tlen.pl (T.G.)

**Keywords:** yerba mate, *Ilex paraguariensis*, phenolic compounds, LC-MS/MS, optimization, extraction

## Abstract

Yerba mate beverage is prepared from *Ilex paraguariensis* grown in South America, and its popularity still is increasing. The aim of this study was to show the differences in the content of antioxidant compounds between hot- and cold-prepared mate beverages. The antioxidant capacity of the infusions was tested using the Folin–Ciocalteu, ABTS, and DPPH assays. A comprehensive analysis of different phenolic and other constituents was performed with the use of high-performance liquid chromatography–tandem mass spectrometry (LC-MS/MS). The hot-prepared infusions on average contain considerably more active compounds than the cold-prepared infusions, which resulted in greater antioxidant activity of hot-prepared beverages. While the hot-prepared infusions of pure yerba mate contained a similar amount of bioactive compounds as those with different additives, for cold-prepared beverages, greater antioxidant activity was found for samples containing additives. In both conditions, the lowest antioxidant activity was noted for *Ilex guayusa*, which contained less rutin, caffeoylquinic acids, dicaffeoylquinic acids, and theobromine, but more caffeine.

## 1. Introduction

Yerba mate is a beverage prepared from the dried leaves and twigs of *Ilex paraguariensis*. This evergreen tree, belonging to the holly family, is found only in South American territories, more specifically in Paraguay, Uruguay, Argentina, and Brazil [1,2,3]. The first harvest of the raw material for the preparation of yerba mate is possible 3–5 years after the plant is planted, and subsequent harvests take place periodically every 3 years. The harvested leaves and twigs undergo a series of processes including roasting, drying, and ripening. The process determines the subsequent properties of the prepared infusion, including, first of all, the taste and content of bioactive compounds [1,3,4].

The consumption of yerba mate is defined by strict rules and has been part of the culture of South American countries for centuries. At present, Argentina is the world’s top producer of yerba mate, accounting for 54% of the production of green leaves, followed by Brazil, which contributes 40%, and Paraguay at 6% [5]. Nevertheless, current consumers are increasingly seeking goods with unique health benefit qualities and refined taste profiles, which has resulted in steadily rising yerba mate sales worldwide [6]. The global yerba mate market is projected to grow from approximately USD 2.09 billion in 2023 to nearly USD 3.28 billion by 2032, representing a compound annual growth rate (CAGR) of 5.15% throughout the forecast period of 2024 to 2032 [7]. In regions where yerba mate grows naturally, the average daily intake of dried leaves is estimated to be around 12–23 g, equating to approximately 1 L of the beverage consumed per day [8]. Depending on the local customs and personal preferences, yerba mate may be prepared in various ways. Most often, dried holly is poured over hot water at 65–95 °C and drank hot with a straw. However, yerba mate can also be prepared with cold water and with the addition of herbs and citrus juice [9]. The rising popularity of this beverage is attributed not only to its sensory qualities but also to its favorable health-promoting properties [10].

Yerba mate is characterized by a multitude of bioactive compounds. The main groups contained in the infusions are purine alkaloids (caffeine, theobromine, and theophylline) [11,12], phenolic compounds (chlorogenic acids, dicaffeoylquinic acids, quinic acid, and rutin) [13,14,15,16], and saponins [1,2,4,17,18]. Due to the high caffeine content of yerba mate, which is similar to that of coffee, it is often consumed for its stimulating properties [1,19]. However, phenolic compounds are mainly responsible for the health benefits of yerba mate consumption, such as antioxidant [20], anti-inflammatory [19], cardioprotective [8], and anti-diabetic [21] effects. Studies have shown the dependence of yerba mate’s antioxidant effects on the polyphenol content of the brew [21,22,23,24]. Among other things, they are responsible for protecting low-density lipoprotein (LDL) from oxidation, thereby protecting against the development of atherosclerosis [24,25,26,27]. In addition, they may contribute to reducing LDL and triglyceride levels in the blood [21,28,29,30]. Consumption of yerba mate has been associated in studies with a reduction in blood glucose levels, an overall improvement in sugar metabolism, and an increase in insulin sensitivity [31,32].

The aim of this study was (1) to optimize the extraction in terms of temperature and time of the process for yerba mate infusions prepared by brewing in both hot and cold water based on the greatest antioxidant activity and total phenolic content, (2) to compare antioxidant activity and total phenolic content between the hot-brewed and cold-brewed yerba mate, and (3) to perform a comprehensive analysis of different phenolic and other constituents present in yerba mate using high-performance liquid chromatography–tandem mass spectrometry.

## 2. Materials and Methods

### 2.1. Chemicals and Samples

2,2′-azinobis(3-ethylbenzothiazoline-6-sulfonic acid) diammonium salt (ABTS), 2,2-diphenyl-1-picrylhydrazyl (DPPH), 6-hydroxy-2,5,7,8-tetramethylchroman-2-carboxylic acid (Trolox), Folin–Ciocalteu reagent, gallic acid, all standards used in LC-MS/MS analysis (Table 1), MS-grade acetonitrile, methanol, and formic acid were purchased from Merck (Steinheim, Germany). Anhydrous sodium carbonate and potassium persulfate were obtained from Avantor Performance Materials Poland S.A. (Gliwice, Poland). High-purity water used in the study was obtained using a reverse osmosis system (DEMIWA 5 ROSA, Watek, Ledeč nad Sázavou, Czech Republic) and double distillation (quartz apparatus, Bi18, Heraeus, Hanau, Germany).

Yerba mate, either pure or with additives, was purchased at a local supermarket (Poznań, Poland) and from the Zielony Bazar internet store (Goczałkowice-Zdrój, Poland) and is described in Table 2.

### 2.2. Sample Preparation

Yerba mate infusions were prepared in two different ways—using cold and warm water as it is made by South Americans who have been drinking yerba for years. Therefore, no organic solvents or ultrasound-assisted extraction were used in optimizing brewing. Both procedures were optimized to reach the highest antioxidant properties of the resulting infusions. Brewing in hot water was optimized using two parameters, temperature (at 60–100 °C) and time (from 2 to 10 min), as presented in Table S1. For this purpose, the central composite design (CCD) was used for the optimization of experiments (the factorial design contained four factorial points, four axial points, and five central points). Brewing in cold water was performed at a constant temperature of 3 °C for 5 to 60 min (5, 10, 20, 30, or 60 min). To optimize the preparation of yerba mate, beverages were performed using sample No. 1 from Paraguay containing 100% *Ilex paraguariensis*.

For preparation of infusions, 25 mL of distilled water was poured onto 1.0 g of samples in an Erlenmeyer flask. After the required extraction time, the infusions were filtrated through type 388, 9 cm diameter filter paper from Filtrak (Bärenstein, Germany). All prepared extracts were analyzed with 3 spectrophotometric tests to show the differences between samples based on their antioxidant activities and the total phenolic compound content. These were the DPPH radical and the ABTS cation radical methods for antioxidant activities and the Folin–Ciocalteu method for total phenolic content.

### 2.3. Methods

To measure the antioxidant activity and total phenolic compounds content of yerba mate infusions, DPPH, ABTS, and Folin–Ciocalteu spectrophotometric methods were used.

#### 2.3.1. DPPH Method

The ability of yerba mate infusions to scavenge DPPH radicals was studied according to the method previously described in [33,34]. The results were expressed as mg of Trolox equivalent to 1 mL of the extract.

#### 2.3.2. ABTS Method

The antioxidant activity of extracts was analyzed with the use of the ABTS cation radical method previously described in [33,35]. The results were expressed as mg of Trolox equivalent to 1 mL of the extract.

#### 2.3.3. Folin–Ciocalteu Method

The total phenolic content was measured with the use of Folin–Ciocalteu reagent according to the method described previously in [33,36]. The results were expressed as mg of gallic acid equivalent (GAE) to 1 mL of the extract. The analyses of the samples measured by all the above methods were performed under dark conditions.

#### 2.3.4. LC-MS/MS Method

The determination of bioactive compounds was carried out using the UltiMate 3000 RSLC chromatographic system from Dionex (Sunnyvale, CA, USA) coupled through the electrospray ionization (ESI) source with the API 4000 QTRAP triple-quadrupole mass spectrometer from AB Sciex (Foster City, CA, USA). The infusion samples were appropriately diluted with 7% acetonitrile in water and filtered with 0.2 µm PTFE syringe filters (Fisherbrand from Argenta, Poznań, Poland) before injecting 5 µL into an Ascentis Express 90A RP-Amide column (100 mm × 2.1 mm I.D.; 2.7 µm) from Supelco, Merck (Darmstadt, Germany), maintained at 35 °C. A gradient of 0.1% formic acid in water (A) and acetonitrile (B) flowing at 0.3 mL min^−1^ was used (0 min, 7% B; 2 min, 15% B; 5 min, 40% B; 6 min, 60% B; 6.5 min, 100% B; 8.6 min, 100% B). The ESI source and mass spectrometer parameters were as follows: curtain gas pressure, 20 psi; nebulizing gas pressure, 45 psi; auxiliary gas pressure, 45 psi; source temperature, 450 °C; ESI voltage, 4500 V; and collision gas set to medium. The dwell time for each transition in the multiple reaction monitoring mode was set to 50 ms. Compound-dependent detection settings are presented in Supplementary Table S2. Quantitation was performed using the external standard method. The limit of detection (LOD) was calculated as 3 times the signal-to-noise ratio and the limit of quantification (LOQ) as 10 times the signal-to-noise ratio. The matrix effect was assessed by dividing slopes obtained for standard curves by those obtained for samples spiked at different concentrations. Values that are greater than 1 indicate signal enhancement, while those that are lower than 1 indicate suppression. Values between 0.8 and 1.2 are considered acceptable [37]. The results are presented in Table S3.

### 2.4. Statistical Analysis

The adequacy of the central composite design models was determined by evaluating the lack of fit, coefficient of determination R^2^, and adjusted R^2^, and the Fisher test value (F value) was determined from the analysis of variance (ANOVA). The experimental data were analyzed using Design Expert ver. 10 software (Stat-Ease Inc., Minneapolis, MN, USA).

## 3. Results

### 3.1. Optimization of the Extraction Process of Yerba Mate Beverages

The optimization results for tested parameters are presented in Tables S4 and S5. For hot brewing optimization, the results obtained in the ABTS and DPPH tests and with the Folin–Ciocalteu reagent were statistically processed. Statistical treatment allowed the models to be fitted to the results from the ABTS test and with the Folin–Ciocalteu reagent (Table S6). In contrast, the model for the test with DPPH was not statistically significant. Based on the results obtained, it can be seen that brewing temperature is weakly correlated with antioxidant activity and total phenolic compounds (Figure S1). What is apparent, however, is the strong relationship between the time of preparation of yerba mate infusions and the results of these tests. Therefore, the longest time used in the optimization study, i.e., 10 min, was adopted for further research, and 80 °C was used as the temperature of the water used for brewing.

For the optimization of low-temperature infusion preparation, the results obtained with all three methods indicated increasing efficiency of extraction of active ingredients as time increased. It was noted that increasing the extraction time to as much as 1 h did not yield results similar to those obtained when brewing in the hot-water experiment. Although the temperature did not play a significant role in the hot preparation of the yerba (at 60 to 100 °C), its reduction to 3 °C results in a noticeable deterioration in the extraction of the active ingredients. For easier comparison of the results obtained when testing the yerba samples, a time equal to the hot-brewing time, i.e., 10 min, was selected. In this way, knowledge will be gained as to which compounds are extracted in significantly smaller quantities under conditions differing only in temperature (80 and 3 °C).

### 3.2. Antioxidant Properties of Yerba Mate Beverages

Thirteen different yerba mate products were tested as presented in Table 2. Two types of infusions were prepared from each of them, a hot-water infusion and a cold-water infusion. As expected, based on the optimization results, greater antioxidant activity in ABTS, and DPPH tests as well as greater total phenolic compound content were observed for the infusions prepared in hot water. However, considerable differences between the products were also observed within both types of infusions (Table 3).

The greatest results within the hot-brewed yerba mate were found for sample No. 1, while the lowest were found for samples No. 7 and No. 8. Two samples, No. 7 and No. 8, are the only ones that contained yerba leaves without stems or dust. The addition of fruit flavoring, etc., has no substantial effect on antioxidant activity compared to the vast majority of pure yerba samples. For the cold-water yerba mate infusions, the same two samples, No. 7 and No. 8, were characterized by the lowest antioxidant activity and total phenolic compound content. However, the greatest results were not observed for sample No. 1 but instead for most samples containing additives. It is interesting to note that the beverage from sample No. 8 containing *Ilex guayusa* has the lowest antioxidant properties under both hot and cold preparation conditions. Moreover, even under hot brewing conditions, its antioxidant activity was lower than that of most cold-prepared beverages. This can be explained by the very low content of rutin, caffeoylquinic acids, and dicaffeoylquinic acids compared to *Ilex paraguariensis*, as shown later in the paper.

### 3.3. Bioactive Compounds in Hot and Cold Yerba Mate Beverages

Yerba mate beverages were prepared using both hot and cold water based on the conditions described above. In both conditions, 13 samples were prepared from materials described in Table 2 and analyzed using LC-MS/MS (Figure S2). The results are given in Table 4, Table 5, Table 6 and Table 7.

Out of 33 compounds determined in the yerba mate beverages, 8 were not detected. These include melatonin, serotonin, myricetin, gallocatechin, catechin gallate, epicatechin gallate, gallocatechin gallate, and epigallocatechin gallate.

The highest level of bioactive compounds, as a total sum of the determined compounds, was found for yerba mate sample No. 10 (leaves from Brazil with the addition of different herbs and also ginkgo biloba leaves), at 2404 µg mL^−1^ prepared with hot-water extraction. The lowest level was found for sample No. 8 (Ecuador *Ilex guayusa*), at 516 µg mL^−1^ (Table 4 and Table 6). For cold beverages, the total sum was on average 2.0 times lower than in hot beverages (Table 5 and Table 7).

The main compound in all hot and cold beverages was 5-caffeyolquinic acid (5-CQA) with two exceptions, and other derivatives of this acid were also determined in high amounts. The second most common was 3-CQA or 3,5-CQA, followed by 4-CQA and 4,5-CQA, and the highest level of the sum of these acids was determined to be 1799 µg mL^−1^ (sample No. 10) in hot beverages and 1155 µg mL^−1^ (sample No. 2) in cold beverages. The lowest level of these acids was found in samples No. 7 and No. 8, at 217 µg mL^−1^ and 149 µg mL^−1^, respectively, and in cold-brewed samples No. 7 and No. 8, at 44 µg mL^−1^ and 57 µg mL^−1^, respectively.

Quinic acid and rutin were also found at high concentrations in all beverages. The level of quinic acid was between 221 and 384 µg mL^−1^ in hot infusions and between 107 and 248 µg mL^−1^ in cold brews. In the case of rutin, the level was lower than quinic acid, i.e., 0.9–152 µg mL^−1^ in hot teas and 0.4–72 µg mL^−1^ in cold teas.

Other phenolics such as caffeic acid, ferulic acid, and sinapic acids and other flavonols such as quercetin and kaempferol were also determined in mate teas in lower amounts. The total level of flavonols in hot teas resulted between 1.0 and 152 µg mL^−1^, while for cold beverages, it was about two times lower: 0.5–72 µg mL^−1^.

Caffeine and theobromine were the dominating alkaloids in all beverages, and the total level of alkaloids (methylxanthines and trigonelline) resulted between 29 and 76 µg mL^−1^ and between 5.9 and 40 µg mL^−1^ in hot- and cold-brewed beverages, respectively. The level of caffeine was determined to be between 10 µg mL^−1^ and 31 µg mL^−1^ for hot beverages and between 1.7 and 13 µg mL^−1^ in cold teas. The highest CAF content under both temperature conditions was recorded for sample No. 8, which included *Ilex guayusa*. Under low-temperature conditions, a similar CAF concentration was obtained for sample No. 10, which additionally contained guarana in its composition. The lowest content of CAF was observed in sample No. 7. The theobromine level was in some cases even higher, and the determined amount was between 4.3 and 51 µg mL^−1^ in hot beverages and between 1.3 and 27 in cold ones. Theophylline was found between 0.10 and 0.33 µg mL^−1^ in hot beverages and lower for cold drinks, at 0.05 to 0.12 µg mL^−1^. In the case of trigonelline, the amount of this alkaloid was low, between 0.013 and 0.168 µg mL^−1^ in hot beverages and between 0.007 and 0.093 µg mL^−1^ in cold beverages.

Nicotinamide was the dominant compound from the amines group, and the level of this compound was between 0.14 and 0.32 µg mL^−1^ for hot beverages and between 0.03 and 0.152 µg mL^−1^ in the case of cold beverages. Theanine’s level was between 0.007 and 0.028 µg mL^−1^ for hot beverages and between 0.006 and 0.022 µg mL^−1^ for cold beverages. The nicotinic acid level was higher than the level of theanine in hot beverages (0.026–0.125 µg mL^−1^) and in most cases higher also in cold beverages (0.011–0.026 µg mL^−1^).

It was found that only two catechins, catechin and epicatechin, were examined in six samples of hot beverages and in five samples of cold beverages with additives.

## 4. Discussion

The first aim of the study was to optimize the extraction process to reflect the greatest antioxidant activity of the yerba mate beverage and the highest level of total phenolic compound content, including phenolic compounds with bioactive activities. The hot optimization extraction process of yerba mate beverages was carried out using water above 60 °C for 10 min to show higher antioxidant activities (Supplementary Figure S1). Interestingly, there was no influence of brewing temperature found.

It is worth mentioning that in this experiment, the results showed significant differences between the hot and cold beverages. The study revealed that lower water temperatures used to extract yerba mate and prepare the drinks resulted in a decrease in the concentration of extracted compounds found in the final beverage. However, even when preparing beverages at cold temperatures, they contain a considerable amount of bioactive compounds and, in addition, can help provide relief during hot days.

For both hot and cold yerba mate beverages, there is a visible tendency for the results of the tests to be increased for longer preparation times. However, when it comes to the daily use of yerba mate, preparing it over a long period of time can be impractical. For hot drinks, it is wise to prepare the brew with boiling water and wait until the temperature of the drink is no higher than 70 °C for consumption (this usually takes about ten minutes). This requirement was recommended by IARC as drinking very hot beverages is probably carcinogenic [38].

Silveira et al. [16] found that using 95 °C water on coarse-ground yerba mate yielded the highest extraction percentage of 24.5% for all compounds. Temperatures of 65 °C, 75 °C, and 85 °C resulted in extraction percentages of 16.2%, 13.2%, and 16.2%, respectively, which were still higher than those from traditional yerba mate (5.9–8.1%). The increase in temperature did not significantly affect the phenolic compound transfer in traditional yerba mate (*p* > 0.05). This may be due to the greater compaction and smaller particle size of traditional yerba mate, reducing extractability and suppressing the impact of infusion temperature.

Kruszewski et al. [39] found that caffeine content in yerba mate increases with longer brewing times, from 15 mg 100 mL^−1^ in 15 min to 25.8 mg 100 mL^−1^ in 60 min at 70 °C. In boiling water (100 °C), it ranges from 11.8 mg 100 mL^−1^ (15 min) to 15.8 mg 100 mL^−1^ (60 min). Their research also shows that infusions brewed at 100 °C have significantly lower caffeine than those at 70 °C, which is confirmed by our findings. Single-brewed yerba mate at 100 °C had a lower caffeine content by 1.87 ± 0.04 mg g^−1^ d.m. (YM-B-1), 2.82 ± 0.75 mg g^−1^ d.m. (YM-A-1), and 6.48 ± 0.71 mg g^−1^ d.m. (YM-C-1) compared to 70 °C. Double-brewed samples showed similar results, with lower caffeine by 2.23 ± 0.04 mg g^−1^ d.m. (YM-B-2), 2.60 ± 0.55 mg g^−1^ d.m. (YM-A-2), and 5.78 ± 1.52 mg g^−1^ d.m. (YM-C-2) at 100 °C compared to 70 °C.

It was also previously found that the results of optimization of chlorogenic acid content (CGA) from yerba mate stems (discarded green stems) showed that the yield and kinetics of solid–liquid extraction of CGA from yerba mate stems were mainly influenced by the solid-to-liquid ratio, number of extractions, temperature, and particle size. Therefore, for yerba mate stems with a particle size diameter < 500 μm, the optimal extraction conditions of CGA (1.051 ± 0.015 mg mL^−1^) at lab scale were to run two-stage extraction at 65 °C using a solid-to-liquid ratio of 1:20 (m:v) [40]. Najman et al. [19] also concluded that infusions prepared at a lower temperature (70 °C in contrast to 100 °C) were characterized by a higher content of total polyphenols and higher antioxidant activity.

In our research, we performed hot extraction at 80 °C in a one-stage extraction process regarding optimization conditions and antioxidant activities of samples.

The next aim of the present study was to determine bioactive compounds with the use of LC-MS/MS in hot and cold yerba mate beverages. The bioactive compounds determined in yerba mate infusions were from different groups: theanine and alkaloids such as trigonelline and methylxanthines (caffeine, theobromine, and theophylline). Phenolic compounds were divided into phenolic acids and complex phenolic compounds such as catechins (catechin and epicatechin) and flavonols such as rutin, quercetin, and kaempferol. Phenolic acids such as ferulic, caffeic, or chlorogenic acids and other acids are also found in yerba mate beverages.

Structurally, chlorogenic acid (CGA; CAS number: 327-97-9) is an ester formed between caffeic and l-quinic acid (5-*O*-caffeoyl-quinic acid). Other isomers of CGA can include 3-*O*-caffeoyl-quinic acid (neochlorogenic acid, neo-CGA) and 4-*O*-caffeoyl-quinic (cryptochlorogenic acid, crypto-CGA). CGA is one of the polyphenol derivatives that is widely present in higher plants [41].

In the present study, it was found that the dominating compounds in both hot and cold yerba mate beverages made from roasted material were caffeoylquinic and dicaffeoylquinic acids. This is similar to previous research where after the roasting process, other compounds were formed from those acids such as caffeoylshikimic acid and dicaffeoylshikimic acid [42]. Previously, it was found that green yerba mate water and ethanolic extracts possessed caffeic acid, quinic acid, caffeoyl glucose, caffeoylquinic acid, feruloylquinic acid, dicaffeoylquinic acid, and rutin [42].

The latest research presented by Rząsa-Duran et al. [12] showed that the dominant compounds were neochlorogenic acid and in one case chlorogenic acids (3-CGA or 4-CGA), which is in opposition to our results. They also found that isochlorogenic acid was determined in a similar amount as neochlorogenic acid. Caffeic acid and caffeine were determined at the same level as in the present research. Similar results for caffeine and theobromine were obtained by Mateos et al. [43].

In a study conducted by Duarte et al., yerba mate leaves with different morphotypes of *Ilex paraguariensis* were extracted at 60 °C using a thermomixer [20]. The results indicated that the caffeine content varies only between the dark green (highest content) and “peludinha” (lowest content) Ilex paraguariensis morphotypes. All five yerba mate morphotypes—“sassafras”, dark green, dull green, gray, and “peludinha”—exhibit similarly high antioxidant capacity and high phenolic compound content. Although the anatomy of the leaf mesophyll is similar, the deposition of wax and cuticle striation on the adaxial surface differs among all five morphotypes.

Caffeine and theobromine were the dominant alkaloids in hot and cold beverages, and trigonelline and theophylline were determined in smaller amounts, more than 100 times lower than caffeine. A cup (about 150 mL) of yerba mate infusion has around 80 mg of caffeine, which is similar to coffee. However, the amount of alkaloids varies based on raw materials and preparation. A study on beverages from Uruguay, Argentina, and Brazil found Uruguayan beverages had the most caffeine and theobromine, followed by Argentine, and Brazilian ones having the least [44].

In another study, it was shown that yerba mate from Argentina, Brazil, Paraguay, and Uruguay contains 100–180 mg g^−1^ of total polyphenols. A teaspoon (5 g) of yerba mate can provide 0.5–0.9 g of polyphenols. Paraguayan plantations produce the richest polyphenolic compounds. Colpo et al. [44] found that Uruguayan brews had 3–5 times more polyphenols in second brews compared to Brazilian ones, with 1451 mg, 967 mg, and 599 mg per 100 mL for Uruguayan, Argentinian, and Brazilian infusions, respectively. Meinhart et al. [45] also noted higher polyphenols in cold-brewed beverages than in hot-water brews.

Zieliński et al. [46] used yerba mate samples from three different Brazilian states (Paraná, Santa Catarina, and Rio Grande do Sul) and water at 80 °C. They used chemometrics to evaluate the antioxidant compounds of yerba mate (*chimarrão*) sold in southern Brazil. Neochlorogenic acid and caffeine were the main bioactive compounds identified. The dataset was analyzed using chemometric techniques. Other studies confirmed these results. The literature indicates that flavonoids are the second most abundant phenolic components in yerba mate, constituting up to 10% of all phenolic compounds [22,43]. It depends on the extraction process and time and other factors as well.

Looking at the results of the LC-MS/MS analysis, it can be concluded that yerba mate contains a large amount of various bioactive compounds. In beverages, caffeoylquinic acids and dicaffeoylquinic acids, particularly chlorogenic acid, quantitatively dominate. Significant amounts of compounds such as quinic acid, caffeic acid, and rutin are also present. As for purine alkaloids, the caffeine content oscillated around 500 μg of extracted compound per gram of dried material. The theobromine content in a significant number of cases was higher than the caffeine content. In some products, it was even twice as much. These results differ from the literature data, which indicate that yerba mate contains more caffeine than theobromine. The last of the purine alkaloids, theophylline, is present in beverages in much smaller amounts. Catechins were detected in only six products, five of which contained additives. These additives are most likely responsible for the presence of catechins in the beverages. The situation was different for gallocatechin, which was present in most samples, but its amount was so small that it could not be quantified. Myricetin was detected only in some samples but not quantified.

Significant differences were visible in the amount of extracted compounds during the preparation of yerba mate in hot and cold water, favoring hot infusions. This method provided up to four times higher concentration in the hot-water extract. However, at low temperatures, kaempferol and catechin were better extractable for some yerba samples, but these differences were not very significant. It was found that samples No. 7 (*Ilex paraguariensis*) and No. 8 (*Ilex guayusa*), both without stems and dust, contained fewer bioactive compounds than the others. The contents of individual bioactive compounds vary greatly between yerba mate products, which may be influenced by the mentioned additives present in the product, the amount of stems and dust, raw material processing conditions, as well as the country of origin and related cultivation conditions of the holly. However, a similar profile of compounds present in yerba mate is maintained for most products prepared hot and cold. Significant differences can be observed in the case of sample No. 8. It did not contain *Ilex paraguariensis* but rather its sister plant, *Ilex guayusa*. The hot-prepared infusions from this product contained the most caffeine, and in the case of cold preparation, only sample No. 10 contained caffeine at the same level. Yerba mate sample No. 10, however, additionally contained guarana, which could influence the higher caffeine content of this product. Guarana possesses a high level of caffeine [47]. The incorporation of ground guarana (*Paullinia cupana*) leaves enhances the stimulating properties of yerba mate by increasing the caffeine content of the brew [12].

Additionally, sample No. 8 was characterized by a low content of compounds such as caffeoylquinic acids and dicaffeoylquinic acids, caffeic acid, rutin, and theobromine. It should be emphasized that these compounds were present in the largest amounts in the rest of the products. Similar levels of these compounds were shown by extracts obtained from yerba mate No. 7. This product had very low contents of RUT, flavonols (QUR and KAE), and flavanols (3-CQ, 4-CQ, 5-CQ, 3,4-DCQ, 3,5-DCQ, and 4,5-DCQ) characteristic of yerba mate. Also, it contained the lowest amount of CAF (in contrast to the *Ilex guayusa* sample). These results raise some doubts about the authenticity of the composition of this product.

Nevertheless, it must be taken into account, that the availability of only one sample of *Ilex guayusa* may be considered a limitation of the study. The difference between *Ilex paraguariensis* and its sister plant *Ilex guayusa* is considerable, but it should be confirmed in another study.

## 5. Conclusions

The preparation of yerba mate beverages was optimized using hot and cold water. Although no considerable temperature influence was found between 60 °C and 100 °C, lower antioxidant properties were obtained at 3 °C. Noticeably, the growth of antioxidant properties with the increase in time was observed for both hot and cold preparation. The final conditions adopted for comparison of different yerba products were 10 min at 80 °C and 10 min at 3 °C.

The antioxidant properties of yerba mate prepared at 80 °C were similar for both pure *Ilex paraguariensis* and *Ilex paraguariensis* containing additives (mainly dried fruits). Interestingly, for cold-prepared yerba mate, greater antioxidant activity was found for samples containing additives.

The compounds found in the highest concentrations in yerba mate were rutin, quinic acid, caffeoylquinic acids, and dicaffeoylquinic acids, which are largely responsible for the antioxidant properties of yerba beverages. On the other hand, the lowest antioxidant activity was noted for *Ilex guayusa*, which contained less rutin, caffeoylquinic acids, and dicaffeoylquinic acids than *Ilex paraguariensis*. Also, it contained more caffeine and less theobromine. Thus, the characteristic profile of compounds in *Ilex paraguariensis* allows us to distinguish it from other products.

## Figures and Tables

**Table 1:** Abbreviations and names of compounds determined in the present study.

Abbreviation	Compound Name	Abbreviation	Compound Name
TRG	Trigonelline	SIN	Synapic acid
NICAM	Nicotinamide	SER	Serotonin
NICAC	Nicotinic acid	MEL	Melatonin
THB	Theobromine	KAE	Kaempferol
THP	Theophylline	SYR	Syringic acid
CAF	Caffeine	SAL	Salicylic acid
THEA	Theanine	RUT	Rutin
3,4-DCQ	3,4-di- caffeoylquinic acid	QUR	Quercetin
3,5-DCQ	3,5-di- caffeoylquinic acid	MYR	Myricetin
4,5-DCQ	4,5-di- caffeoylquinic acid	C	Catechin
4-CQ	4- caffeoylquinic acid	EC	Epicatechin
3-CQ	3- caffeoylquinic acid	GC	Gallocatechin
5-CQ	5- caffeoylquinic acid	CG	Catechin gallate
CAFAC	Caffeic acid	GCG	Gallocatechin gallate
COU	Coumaric acid	EGCG	Epigallocatechin gallate
FER	Ferulic acid	ECG	Epicatechin gallate
QUI	Quinic acid		

**Table 2:** Yerba mate used in experiments.

No.	Origin	Ingredients
1	Paraguay	100% *Ilex paraguariensis* (leaves and stems with low level of dust)
2	Paraguay	100% *Ilex paraguariensis* (leaves and stems with low level of dust)
3	Paraguay	100% *Ilex paraguariensis* (leaves and stems with low level of dust)
4	Paraguay	100% *Ilex paraguariensis* (leaves with stems and dust)
5	Argentina	100% *Ilex paraguariensis* (leaves and stems with medium level of dust)
6	Brazil	100% *Ilex paraguariensis* (leaves and stems with medium level of dust)
7	Brazil	100% *Ilex paraguariensis* (leaves without stems)
8	Ecuador	100% *Ilex guayusa* (leaves without stems)
9	Brazil	97.8% *Ilex paraguariensis* (leaves and stems with medium level of dust), ground guarana, and natural flavoring
10	Brazil	94.5% *Ilex paraguariensis* (leaves without dust), guarana, pineapple, orange skin, ginseng, ginkgo biloba, cornflower petals, and natural mango and orange flavoring
11	Brazil	93.9% *Ilex paraguariensis* (leaves without stems), dried apple, cinnamon, natural clove oil, and natural flavoring
12	Brazil	91.2% *Ilex paraguariensis* (leaves and stems with medium level of dust), apple, papaya, mint, and natural flavoring
13	Brazil	91.1% *Ilex paraguariensis* (leaves and stems with low level of dust), lemon grass, mint, lemon verbena, 1% wormwood herb, 1% hemp flour, and natural flavoring

**Table 3:** Results from the Folin–Ciocalteu, ABTS, and DPPH tests obtained for yerba mate beverages prepared in hot and cold water (mean ± standard deviation).

Sample	Preparation Conditions	Folin–Ciocalteu [mg GAE mL^−1^]	ABTS [mg Trolox mL^−1^]	DPPH [mg Trolox mL^−1^]
1	10 min at 80 °C	3.959 ± 0.198	4.079 ± 0.163	4.776 ± 0.191
2		2.749 ± 0.165	3.233 ± 0.162	3.803 ± 0.190
3		2.081 ± 0.104	2.175 ± 0.152	2.583 ± 0.077
4		1.998 ± 0.100	2.244 ± 0.135	2.700 ± 0.162
5		2.515 ± 0.126	2.056 ± 0.123	2.013 ± 0.060
6		1.928 ± 0.116	1.845 ± 0.129	2.396 ± 0.120
7		1.143 ± 0.057	1.310 ± 0.052	1.320 ± 0.079
8		0.900 ± 0.045	0.841 ± 0.034	0.973 ± 0.039
9		1.993 ± 0.100	2.115 ± 0.106	2.529 ± 0.126
10		2.790 ± 0.139	2.529 ± 0.076	2.854 ± 0.143
11		2.504 ± 0.125	2.909 ± 0.087	3.118 ± 0.094
12		2.139 ± 0.107	2.420 ± 0.097	2.424 ± 0.145
13		2.561 ± 0.077	2.772 ± 0.111	2.977 ± 0.089
1	10 min at 3 °C	1.077 ± 0.075	1.030 ± 0.062	1.075 ± 0.043
2		1.249 ± 0.050	1.377 ± 0.041	1.297 ± 0.039
3		0.818 ± 0.033	1.044 ± 0.042	1.010 ± 0.071
4		0.901 ± 0.036	1.062 ± 0.064	1.074 ± 0.032
5		0.623 ± 0.019	0.732 ± 0.051	0.791 ± 0.055
6		0.764 ± 0.023	0.857 ± 0.043	0.853 ± 0.026
7		0.230 ± 0.016	0.317 ± 0.016	0.396 ± 0.020
8		0.360 ± 0.022	0.634 ± 0.019	0.490 ± 0.029
9		1.160 ± 0.035	1.338 ± 0.054	1.406 ± 0.084
10		1.448 ± 0.058	1.549 ± 0.108	1.513 ± 0.076
11		1.249 ± 0.050	1.377 ± 0.041	1.297 ± 0.039
12		0.818 ± 0.033	1.044 ± 0.042	1.010 ± 0.071
13		1.239 ± 0.062	1.330 ± 0.080	1.284 ± 0.039

Heatmap colors indicate values from lowest (green) to highest (red).

**Table 4:** Content of amines, vitamin B_3_, alkaloids, flavonols, and flavanols in yerba mate beverages prepared in hot water, µg mL^−1^ ± standard deviation.

Sample	THEA	NICAM	NICAC	TRG	THP	THB	CAF	RUT	QUR	KAE	C	EC
1	0.017 ± 0.001	0.290 ± 0.020	0.030 ± 0.002	0.014 ± 0.001	0.153 ± 0.008	21.28 ± 0.64	21.09 ± 0.63	110.1 ± 4.4	0.052 ± 0.002	0.030 ± 0.001	<LOD	<LOD
2	0.013 ± 0.001	0.297 ± 0.009	0.040 ± 0.003	0.036 ± 0.002	0.195 ± 0.006	21.09 ± 1.48	22.02 ± 0.66	122.8 ± 6.1	0.150 ± 0.007	0.023 ± 0.001	<LOD	<LOD
3	0.015 ± 0.001	0.190 ± 0.006	0.028 ± 0.002	0.013 ± 0.000	0.133 ± 0.004	16.06 ± 0.48	16.66 ± 0.67	82.79 ± 3.31	0.050 ± 0.002	0.010 ± 0.001	<LOD	<LOD
4	0.011 ± 0.001	0.163 ± 0.010	0.026 ± 0.002	0.021 ± 0.001	0.103 ± 0.006	14.15 ± 0.99	14.27 ± 0.86	70.41 ± 2.82	0.047 ± 0.002	0.027 ± 0.001	0.06 ± 0.003	0.11 ± 0.01
5	0.020 ± 0.001	0.185 ± 0.007	0.030 ± 0.002	0.043 ± 0.003	0.221 ± 0.009	35.65 ± 1.78	18.62 ± 0.74	94.24 ± 5.65	0.045 ± 0.001	0.020 ± 0.001	<LOD	<LOD
6	0.023 ± 0.001	0.186 ± 0.006	0.026 ± 0.001	0.029 ± 0.001	0.132 ± 0.008	30.06 ± 0.90	16.39 ± 0.66	80.16 ± 5.61	0.036 ± 0.001	0.028 ± 0.002	<LOD	<LOD
7	0.007 ± 0.001	0.137 ± 0.007	0.125 ± 0.007	0.149 ± 0.006	0.257 ± 0.018	22.95 ± 1.38	10.40 ± 0.31	0.86 ± 0.06	0.062 ± 0.004	0.039 ± 0.001	<LOD	<LOD
8	0.026 ± 0.001	0.137 ± 0.004	0.049 ± 0.002	0.013 ± 0.001	0.330 ± 0.017	4.32 ± 0.17	31.36 ± 1.88	3.92 ± 0.20	<LOD	0.014 ± 0.001	<LOD	<LOD
9	0.023 ± 0.002	0.187 ± 0.009	0.026 ± 0.001	0.049 ± 0.001	0.187 ± 0.013	37.20 ± 1.49	23.08 ± 1.62	103.6 ± 6.2	0.050 ± 0.003	0.013 ± 0.001	1.55 ± 0.06	2.70 ± 0.16
10	0.015 ± 0.001	0.318 ± 0.016	0.037 ± 0.003	0.062 ± 0.002	0.249 ± 0.007	50.80 ± 1.52	24.26 ± 1.70	151.9 ± 6.1	0.102 ± 0.005	0.017 ± 0.001	1.01 ± 0.06	1.77 ± 0.09
11	0.015 ± 0.001	0.207 ± 0.012	0.030 ± 0.001	0.072 ± 0.004	0.210 ± 0.010	34.89 ± 2.44	21.68 ± 1.08	127.2 ± 5.1	0.091 ± 0.003	0.019 ± 0.001	0.03 ± 0.001	0.22 ± 0.01
12	0.028 ± 0.001	0.180 ± 0.009	0.028 ± 0.001	0.057 ± 0.002	0.200 ± 0.006	38.54 ± 1.16	22.15 ± 1.55	106.4 ± 5.3	0.092 ± 0.006	0.028 ± 0.002	<LOQ	0.02 ± 0.001
13	0.014 ± 0.000	0.211 ± 0.013	0.035 ± 0.002	0.168 ± 0.005	0.227 ± 0.009	37.45 ± 1.87	22.05 ± 1.10	137.9 ± 5.5	0.134 ± 0.007	0.044 ± 0.003	0.03 ± 0.002	0.05 ± 0.003

Heatmap colors indicate values from lowest (green) to highest (orange).

**Table 5:** Content of amines, vitamin B_3_, alkaloids, flavonols, and flavanols in yerba mate beverages prepared in cold water, µg mL^−1^ ± standard deviation.

Sample	THEA	NICAM	NICAC	TRG	THP	THB	CAF	RUT	QUR	KAE	C	EC
1	0.013 ± 0.001	0.126 ± 0.005	0.016 ± 0.001	0.011 ± 0.001	0.081 ± 0.003	10.41 ± 0.31	11.24 ± 0.79	57.24 ± 1.72	0.023 ± 0.001	0.035 ± 0.002	<LOD	<LOD
2	0.012 ± 0.001	0.149 ± 0.010	0.026 ± 0.002	0.030 ± 0.002	0.108 ± 0.004	12.33 ± 0.49	12.21 ± 0.61	72.14 ± 3.61	0.036 ± 0.003	0.011 ± 0.001	<LOD	<LOQ
3	0.013 ± 0.000	0.136 ± 0.008	0.020 ± 0.001	0.017 ± 0.001	0.092 ± 0.006	11.22 ± 0.67	11.58 ± 0.58	65.80 ± 1.97	0.032 ± 0.002	0.008 ± 0.001	<LOQ	<LOD
4	0.012 ± 0.001	0.124 ± 0.007	0.018 ± 0.001	0.013 ± 0.001	0.088 ± 0.004	9.01 ± 0.36	9.70 ± 0.58	52.34 ± 2.09	0.030 ± 0.001	0.026 ± 0.001	0.14 ± 0.01	0.18 ± 0.01
5	0.012 ± 0.000	0.081 ± 0.006	0.014 ± 0.001	0.015 ± 0.001	0.074 ± 0.002	12.16 ± 0.36	6.56 ± 0.20	31.93 ± 1.92	0.013 ± 0.001	0.026 ± 0.001	<LOD	<LOD
6	0.020 ± 0.001	0.091 ± 0.003	0.011 ± 0.001	0.013 ± 0.001	0.049 ± 0.003	11.22 ± 0.67	6.63 ± 0.46	31.03 ± 1.24	0.010 ± 0.001	0.007 ± 0.001	<LOQ	<LOD
7	0.006 ± 0.001	0.030 ± 0.002	0.023 ± 0.001	0.056 ± 0.003	0.047 ± 0.003	4.07 ± 0.16	1.71 ± 0.07	0.40 ± 0.02	0.011 ± 0.001	0.027 ± 0.002	<LOD	<LOD
8	0.019 ± 0.001	0.057 ± 0.002	0.019 ± 0.001	0.007 ± 0.001	0.101 ± 0.007	1.33 ± 0.09	12.69 ± 0.63	1.22 ± 0.09	<LOD	0.008 ± 0.001	<LOD	<LOD
9	0.018 ± 0.001	0.111 ± 0.004	0.015 ± 0.001	0.026 ± 0.001	0.094 ± 0.003	18.35 ± 1.28	11.88 ± 0.59	51.00 ± 3.57	0.027 ± 0.001	0.010 ± 0.001	1.70 ± 0.10	2.25 ± 0.11
10	0.011 ± 0.001	0.152 ± 0.009	0.026 ± 0.002	0.042 ± 0.003	0.120 ± 0.008	26.67 ± 1.07	12.91 ± 0.90	70.67 ± 2.12	0.022 ± 0.001	0.012 ± 0.001	1.03 ± 0.05	1.27 ± 0.05
11	0.009 ± 0.001	0.130 ± 0.004	0.018 ± 0.001	0.053 ± 0.002	0.098 ± 0.007	16.45 ± 0.66	10.43 ± 0.31	55.55 ± 2.78	0.043 ± 0.001	0.012 ± 0.001	0.02 ± 0.001	0.14 ± 0.01
12	0.022 ± 0.001	0.111 ± 0.008	0.017 ± 0.001	0.043 ± 0.001	0.079 ± 0.003	16.95 ± 0.51	9.84 ± 0.59	49.81 ± 3.49	0.030 ± 0.001	0.029 ± 0.001	<LOD	<LOQ
13	0.016 ± 0.001	0.128 ± 0.008	0.023 ± 0.001	0.093 ± 0.007	0.104 ± 0.006	17.43 ± 0.70	10.81 ± 0.32	56.13 ± 1.68	0.033 ± 0.001	0.014 ± 0.001	0.02 ± 0.001	<LOQ

Heatmap colors indicate values from lowest (green) to highest (orange).

**Table 6:** Content of phenolic acids and other organic acids in yerba mate beverages prepared in hot water, µg mL^−1^ ± standard deviation.

Sample	SIN	CAFAC	FER	SYR	COU	SAL	QUI	3-CQ	4-CQ	5-CQ	3,4-DCQ	3,5-DCQ	4,5-DCQ
1	0.066 ± 0.003	5.49 ± 0.27	0.159 ± 0.006	0.180 ± 0.009	0.092 ± 0.003	0.171 ± 0.009	276 ± 19	427 ± 17	230 ± 12	528 ± 37	82.0 ± 4.9	326 ± 10	156 ± 9
2	0.075 ± 0.003	5.84 ± 0.41	0.183 ± 0.007	0.129 ± 0.009	0.116 ± 0.008	0.212 ± 0.013	342 ± 14	386 ± 15	232 ± 12	506 ± 25	103 ± 5	349 ± 14	147 ± 7
3	0.049 ± 0.002	3.84 ± 0.12	0.128 ± 0.005	0.119 ± 0.004	0.072 ± 0.002	0.116 ± 0.008	221 ± 9	296 ± 9	151 ± 9	391 ± 12	66.4 ± 4.0	305 ± 12	116 ± 5
4	0.039 ± 0.002	3.38 ± 0.14	0.103 ± 0.003	0.118 ± 0.005	0.068 ± 0.004	0.160 ± 0.005	224 ± 13	310 ± 12	159 ± 10	368 ± 11	73.3 ± 5.1	213 ± 13	101 ± 4
5	0.024 ± 0.001	2.04 ± 0.14	0.097 ± 0.004	0.055 ± 0.003	0.067 ± 0.004	0.172 ± 0.005	268 ± 16	294 ± 9	172 ± 10	445 ± 18	45.7 ± 2.3	207 ± 12	111 ± 7
6	0.023 ± 0.002	1.54 ± 0.08	0.098 ± 0.005	0.051 ± 0.002	0.047 ± 0.002	0.161 ± 0.006	279 ± 17	276 ± 19	137 ± 10	451 ± 23	19.7 ± 1.4	133 ± 8	47.9 ± 1.9
7	0.032 ± 0.001	0.79 ± 0.02	0.344 ± 0.014	0.332 ± 0.023	0.346 ± 0.021	1.368 ± 0.041	384 ± 15	97.0 ± 5.8	50.7 ± 3.5	41.9 ± 1.7	5.0 ± 0.2	10.1 ± 0.3	12.1 ± 0.5
8	0.010 ± 0.000	0.20 ± 0.01	0.045 ± 0.003	0.012 ± 0.001	0.056 ± 0.003	0.173 ± 0.005	326 ± 13	39.5 ± 2.4	16.9 ± 1.0	56.1 ± 2.2	1.2 ± 0.1	30.6 ± 1.5	4.9 ± 0.2
9	0.111 ± 0.008	2.84 ± 0.14	0.125 ± 0.004	0.054 ± 0.002	0.061 ± 0.002	0.136 ± 0.004	280 ± 17	319 ± 16	144 ± 4	508 ± 20	59.4 ± 3.0	306 ± 15	94.8 ± 6.6
10	0.107 ± 0.007	5.70 ± 0.17	0.219 ± 0.007	0.031 ± 0.002	0.084 ± 0.003	0.141 ± 0.004	368 ± 26	330 ± 13	181 ± 9	713 ± 21	88.1 ± 6.2	355 ± 25	132 ± 5
11	0.073 ± 0.005	4.40 ± 0.26	0.170 ± 0.012	0.036 ± 0.001	0.063 ± 0.002	0.131 ± 0.008	311 ± 16	330 ± 10	190 ± 13	579 ± 41	79.8 ± 3.2	316 ± 16	127 ± 5
12	0.112 ± 0.004	3.60 ± 0.22	0.176 ± 0.007	0.060 ± 0.002	0.068 ± 0.004	0.141 ± 0.008	334 ± 23	339 ± 24	172 ± 5	573 ± 40	59.6 ± 3.6	279 ± 17	112 ± 3
13	0.086 ± 0.005	4.53 ± 0.27	0.254 ± 0.008	0.051 ± 0.002	0.091 ± 0.003	0.157 ± 0.011	327 ± 20	348 ± 24	193 ± 12	612 ± 18	87.4 ± 5.2	303 ± 15	123 ± 7

Heatmap colors indicate values from lowest (green) to highest (red).

**Table 7:** Content of phenolic acids and other organic acids in yerba mate beverages prepared in cold water, µg mL^−1^ ± standard deviation.

Sample	SIN	CAFAC	FER	SYR	COU	SAL	QUI	3-CQ	4-CQ	5-CQ	3,4-DCQ	3,5-DCQ	4,5-DCQ
1	0.035 ± 0.001	2.75 ± 0.16	0.084 ± 0.003	0.089 ± 0.004	0.047 ± 0.002	0.098 ± 0.006	173 ± 9	246 ± 15	133 ± 5	303 ± 15	37.4 ± 1.9	147 ± 10	63 ± 4
2	0.044 ± 0.003	3.21 ± 0.16	0.101 ± 0.005	0.080 ± 0.005	0.071 ± 0.003	0.139 ± 0.010	243 ± 17	283 ± 14	163 ± 8	383 ± 15	44.6 ± 2.2	196 ± 6	85 ± 3
3	0.042 ± 0.002	2.63 ± 0.11	0.092 ± 0.005	0.090 ± 0.005	0.051 ± 0.004	0.127 ± 0.005	217 ± 11	273 ± 11	148 ± 9	388 ± 27	44.6 ± 3.1	167 ± 12	74 ± 4
4	0.032 ± 0.002	2.00 ± 0.08	0.072 ± 0.003	0.077 ± 0.005	0.046 ± 0.002	0.116 ± 0.008	183 ± 9	223 ± 11	140 ± 8	304 ± 12	37.1 ± 1.1	147 ± 9	66 ± 5
5	0.018 ± 0.001	0.81 ± 0.06	0.042 ± 0.002	0.028 ± 0.001	0.027 ± 0.001	0.076 ± 0.004	122 ± 6	106 ± 4	58.3 ± 3.5	175 ± 12	15.5 ± 0.9	70.2 ± 2.8	34.3 ± 1.4
6	0.093 ± 0.005	0.75 ± 0.03	0.045 ± 0.003	0.026 ± 0.001	0.019 ± 0.001	0.072 ± 0.003	139 ± 4	121 ± 4	62.3 ± 3.7	203 ± 12	19.1 ± 1.1	91.6 ± 4.6	30.3 ± 1.5
7	<LOQ	0.18 ± 0.01	0.081 ± 0.003	0.077 ± 0.003	0.109 ± 0.003	0.113 ± 0.005	107 ± 3	18.5 ± 0.9	10.9 ± 0.4	9.6 ± 0.6	1.1 ± 0.1	2.1 ± 0.1	2.0 ± 0.1
8	<LOD	0.06 ± 0.00	0.020 ± 0.001	0.005 ± 0.0004	0.021 ± 0.001	0.053 ± 0.002	168 ± 8	16.5 ± 0.5	7.0 ± 0.2	23.5 ± 0.7	0.3 ± 0.0	9.3 ± 0.4	0.6 ± 0.0
9	0.030 ± 0.002	1.64 ± 0.11	0.072 ± 0.004	0.032 ± 0.002	0.033 ± 0.001	0.086 ± 0.004	216 ± 11	179 ± 7	92.4 ± 5.5	327 ± 13	24.3 ± 1.5	142 ± 6	45.0 ± 1.8
10	0.054 ± 0.003	2.98 ± 0.09	0.119 ± 0.006	0.019 ± 0.001	0.045 ± 0.002	0.109 ± 0.008	248 ± 7	199 ± 12	103 ± 5	406 ± 12	33.6 ± 1.0	166 ± 12	49.8 ± 2.0
11	0.081 ± 0.002	2.06 ± 0.06	0.092 ± 0.006	0.020 ± 0.001	0.034 ± 0.002	0.083 ± 0.004	212 ± 11	176 ± 12	97.4 ± 5.8	315 ± 22	27.7 ± 1.1	129 ± 9	45.6 ± 3.2
12	0.109 ± 0.005	1.45 ± 0.04	0.082 ± 0.005	0.032 ± 0.002	0.033 ± 0.002	0.095 ± 0.006	192 ± 12	154 ± 9	78.7 ± 2.4	276 ± 19	28.9 ± 1.4	144 ± 6	46.9 ± 1.9
13	0.033 ± 0.001	2.03 ± 0.10	0.125 ± 0.009	0.028 ± 0.002	0.043 ± 0.003	0.114 ± 0.006	235 ± 9	182 ± 11	93.8 ± 3.8	342 ± 20	26.6 ± 0.8	132 ± 4	42.0 ± 2.5

Heatmap colors indicate values from lowest (green) to highest (red).

## Data Availability

The original contributions presented in the study are included in the article/Supplementary Material, further inquiries can be directed to the corresponding author.

## Supplementary Materials

The following supporting information can be downloaded at: https://www.mdpi.com/article/10.3390/antiox13121467/s1. Figure S1: The response surfaces in the tests (a) Folin–Ciocalteu, (b) ABTS. Parameter A—temperature; parameter B—time. Figure S2. Chromatograms of (a) standard solution, (b) sample 10, (c) sample 8. Table S1: Experimental parameters for optimization of brewing yerba mate in hot water. Table S2: Mass spectrometer parameters applied for the determination of compounds. Table S3: LC-MS/MS parameters for determined compounds (RT—retention time; LOD—limit of detection; LOQ—limit of quantification). Table S4: Results for testing the optimization of the hot brewing extraction. Table S5: Results for testing the optimization of the cold brewing extraction. Table S6: Optimization of model fitting to the experimental results in hot brewing extraction.
