# Supplementary material for: Antioxidant Capacity, Phenolic Compounds, and Other Constituents of Cold and Hot Yerba Mate (Ilex paraguariensis) Infusions
Source: Antioxidants (Basel). 2024 Nov 29;13(12):1467. doi: 10.3390/antiox13121467 (PMC11673172; doi:10.3390/antiox13121467)
Supplement: Supplementary file 1 [file antioxidants-13-01467-s001.zip › Supplement.pdf]

Supplement

Antioxidant Capacity, Phenolic Compounds, and Other Constituents of Cold and Hot Yerba Mate (*Ilex paraguariensis*) Infusions

Julia Płatkiewicz, Daniel Okołowicz, Robert Frankowski, Tomasz Grześkowiak, Magdalena Jeszka-Skowron, and Agnieszka Zgoła-Grześkowiak

Table S1. Experimental parameters for optimization of brewing yerba mate in hot water

Experiment	Parameters	
	Temperature [°C]	Time [min]
1	60	2
2	100	2
3	60	10
4	100	10
5	70	4
6	90	4
7	70	8
8	90	8
9	80	6
10	80	6
11	80	6
12	80	6
13	80	6

Table S2. Mass spectrometer parameters applied for the determination of compounds

Compound	Abbr.	DP[a] [V]	Analytical transition	CE[b] [eV]	EP[c] [V]	CXP[d] [V]	Confirmatory transition	CE [eV]	EP [V]	CXP [V]
Trigonelline	TRG	50	138 → 94	30	10	4	138 → 110	30	10	5
Theanine	THEA	35	175 → 84	30	10	4	175 → 158	17	10	9
Quinic acid	QUI	60	193 → 147	13	5	9	193 → 111	21	5	5
Nicotinamide	NICAM	20	123 → 80	28	10	2	123 → 96	28	10	4
Serotonin	SER	50	177 → 160	9	10	12	177 → 132	31	6	7
Nicotinic acid	NICAC	30	124 → 80	30	10	3	124 → 78	32	10	3
Theobromine	THB	40	181 → 138	25	10	7	181 → 110	31	10	5
Theophylline	THP	50	181 → 124	27	10	6	181 → 96	33	10	4
(-)-Gallocatechin	GC	45	307 → 139	21	3	12	307 → 151	15	3	8
Chlorogenic acid	5-CQ	50	355 → 163	18	5	10	355 → 145	45	3	7
Caffeine	CAF	25	195 → 138	26	10	5	195 → 110	31	10	5
4-O-Caffeoylquinic acid	4-CQ	50	355 → 163	18	5	10	355 → 145	45	3	7
(+)-Catechin	C	70	291 → 139	20	2	6	291 → 165	17	7	9
3-O-Caffeoylquinic acid	3-CQ	50	355 → 163	18	5	10	355 → 145	45	3	7
Syringic acid	SYR	50	199 → 140	21	10	7	199 → 155	15	4	9
(-)-Epicatechin	EC	70	291 → 139	20	2	6	291 → 165	17	7	9
Caffeic acid	CAFAC	48	181 → 163	45	8	9	181 → 135	27	6	7
(-)-Epigallocatechin 3-gallate	EGCG	60	459 → 139	27	3	7	459 → 289	13	4	6
(-)-Gallocatechin 3-gallate	GCG	60	459 → 139	27	3	7	459 → 289	13	4	6
Rutin	RUT	70	611 → 303	28	10	6	611 → 465	18	10	7
Sinapic acid	SIN	40	225 → 207	14	5	4	225 → 175	21	3	15
Melatonin	MEL	85	233 → 174	19	5	10	233 → 159	40	6	10
Ferulic acid	FER	45	195 → 177	15	4	10	195 → 145	23	7	7

(-)-Epicatechin-3-gallate	ECG	60	443 → 123	21	3	6	443 → 273	12	4	6
p-Coumaric acid	COU	45	165 → 147	15	3	7	165 → 119	25	4	6
3,4-Dicaffeoylquinic acid	3,4-DCQ	55	517 → 163	26	5	11	517 → 499	13	5	15
(-)-Catechin 3-gallate	CG	60	443 → 123	21	3	6	443 → 273	12	4	6
3,5-Dicaffeoylquinic acid	3,5-DCQ	55	517 → 163	26	5	11	517 → 499	13	5	15
4,5-Dicaffeoylquinic acid	4,5-DCQ	55	517 → 163	26	5	11	517 → 499	13	5	15
Salicylic acid	SAL	45	139 → 121	18	6	6	139 → 65	35	5	11
Myricetin	MYR	80	319 → 153	44	4	8	319 → 245	40	4	5
Quercetin	QUR	70	303 → 153	47	10	8	303 → 229	41	10	14
Kaempferol	KAE	50	287 → 153	45	5	8	287 → 121	44	5	6

[a]DP – declustering potential, [b]CE – collision energy, [c]EP – cell entrance potential, [d]CXP – cell exit potential

Table S3. LC-MS/MS parameters for determined compounds (RT – retention time; LOD – limit of detection; LOQ – limit of quantification)

Compound	RT [min]	Intercept	Slope	R^2	Range [μg L^{-1}]		LOD [μg L^{-1}]	LOQ [μg L^{-1}]	Matrix effect
					min	max			
Trigonelline	0.80	9220	18190716	0.9994	0.25	50	0.04	0.13	1.05
Theanine	0.82	8021	16453329	0.9989	0.2	100	0.03	0.11	0.95
Quinic acid	0.83	6631	431224	0.9993	20	1000	3.30	11.0	1.18
Nicotinamide	0.87	5165	22713294	0.9994	0.1	25	0.02	0.05	1.16
Serotonin	0.95	7344	9390678	0.9996	1	100	0.02	0.06	1.03
Nicotinic acid	0.99	2930	19350246	0.9998	0.25	25	0.06	0.19	0.99
Theobromine	1.50	4145	6773447	0.9999	0.5	500	0.04	0.12	0.98
Theophylline	2.10	3213	161604129	1.0000	1	100	0.02	0.07	1.12
(-)-Gallocatechin	2.45	-88127	3049944	0.9997	50	1000	0.40	1.33	0.85
Chlorogenic acid	2.63	2948	3868801	0.9999	2	1000	0.29	0.95	1.16
Caffeine	2.72	41193	91746500	0.9994	0.1	50	0.01	0.04	0.93
4-O-Caffeoylquinic acid	3.46	-12615	3627358	0.9995	2	500	0.37	1.23	1.14
(+)-Catechin	3.60	-5251	3403431	0.9999	2	1000	0.31	1.04	1.16
3-O-Caffeoylquinic acid	3.74	-16692	9188853	1.0000	1	500	0.16	0.54	1.04
Syringic acid	3.94	12638	4164220	0.9992	0.5	500	0.07	0.24	1.03
(-)-Epicatechin	3.99	-24444	381349	0.9990	10	1000	0.51	1.30	0.88
Caffeic acid	4.35	-7967	7076998	0.9998	2.5	500	0.31	1.03	1.06
(-)-Epigallocatechin 3-gallate	4.62	-286351	4496598	0.9954	100	1000	1.36	4.53	1.08
(-)-Gallocatechin 3-gallate	4.87	-282366	3902906	0.9984	100	1000	2.55	8.38	1.05
Rutin	4.91	5169	3396976	0.9999	1	500	0.06	0.21	0.82
Sinapic acid	4.94	1384	17126760	0.9999	1	100	0.16	0.53	0.88
Melatonin	5.08	2105	94797110	0.9999	0.02	10	0.0024	0.0079	0.82

Ferulic acid	5.13	35720	11691328	0.9996	2.5	250	0.34	1.15	0.92
(-)-Epicatechin-3-gallate	5.16	-59146	2410929	0.9995	50	1000	0.42	1.40	1.02
p-Coumaric acid	5.34	25484	10673657	0.9997	0.5	500	0.08	0.28	0.94
3,4-Dicaffeoylquinic acid	5.37	-15145	2821934	0.9992	2	500	0.43	1.42	1.01
(-)-Catechin 3-gallate	5.41	-88127	3049944	0.9997	50	1000	0.40	1.33	0.82
3,5-Dicaffeoylquinic acid	5.55	2755	2564587	0.9994	2	500	0.37	1.23	1.03
4,5-Dicaffeoylquinic acid	5.73	-3059	3695709	0.9997	2.5	500	0.36	1.18	1.15
Salicylic acid	6.30	38981	5995791	0.9997	2	100	0.46	1.55	1.04
Myricetin	6.39	-136538	2898150	1.0000	50	250	0.36	1.19	0.88
Quercetin	7.17	-15312	3305479	1.0000	5	50	0.13	0.42	1.06
Kaempferol	7.65	-5672	5226158	0.9999	100	250	0.08	0.25	0.86

Table S4. Results for testing the optimization of the hot brewing extraction

Experiment	Parameters		Results		
	Temperature [°C]	Time [min]	F-C* [mg GAE mL^{-1}]	ABTS [mg Trolox mL^{-1}]	DPPH [mg Trolox mL^{-1}]
1	60	2	1.943	1.556	3.962
2	100	2	2.475	2.427	5.400
3	60	10	2.615	3.119	5.717
4	100	10	2.871	3.140	5.160
5	70	4	2.215	2.307	4.023
6	90	4	2.010	1.989	4.190
7	70	8	2.485	2.244	4.860
8	90	8	1.968	1.762	4.046
9	80	6	2.253	2.011	4.691
10	80	6	2.427	1.738	4.813
11	80	6	2.444	2.334	4.813
12	80	6	2.157	2.535	4.661
13	80	6	2.436	2.487	4.609

* The Folin-Ciocalteu's method results

Table S5. Results for testing the optimization of the cold brewing extraction

Experiment	Parameters		Results		
	Temperature [°C]	Time [min]	F-C* [mg GAE mL^{-1}]	ABTS [mg Trolox mL^{-1}]	DPPH [mg Trolox mL^{-1}]
1	3	5	1.378	1.597	3.290
2	3	10	1.291	1.582	2.454
3	3	20	1.471	1.719	2.966
4	3	30	1.585	1.974	3.150
5	3	60	2.044	2.479	3.737

* The Folin-Ciocalteu's method results

Table S6. Optimization of model fitting to the experimental results in hot brewing extraction

Parameters	Model for the Folin-Ciocalteu test			Model for the ABTS test		
	F value	P value	Importance	F value	P value	Importance
Model	5.0715	0.0457	significant	5.2200	0.0431	significant
B: Time	5.0715	0.0457	significant	5.2200	0.0431	significant
Lack of fit	4.4514	0.0838	non-significant	3.3510	0.1298	non-significant

a)

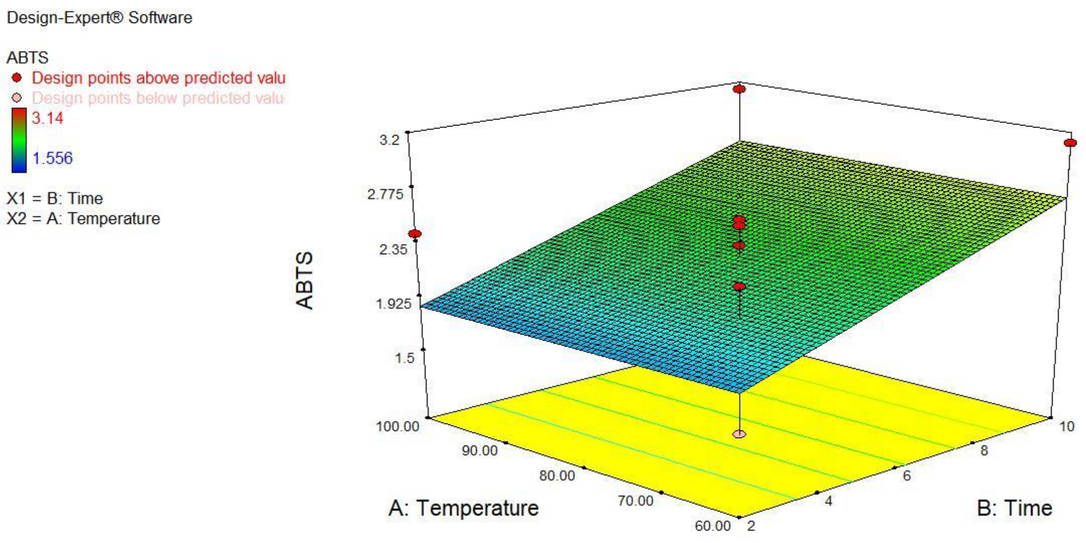

b)

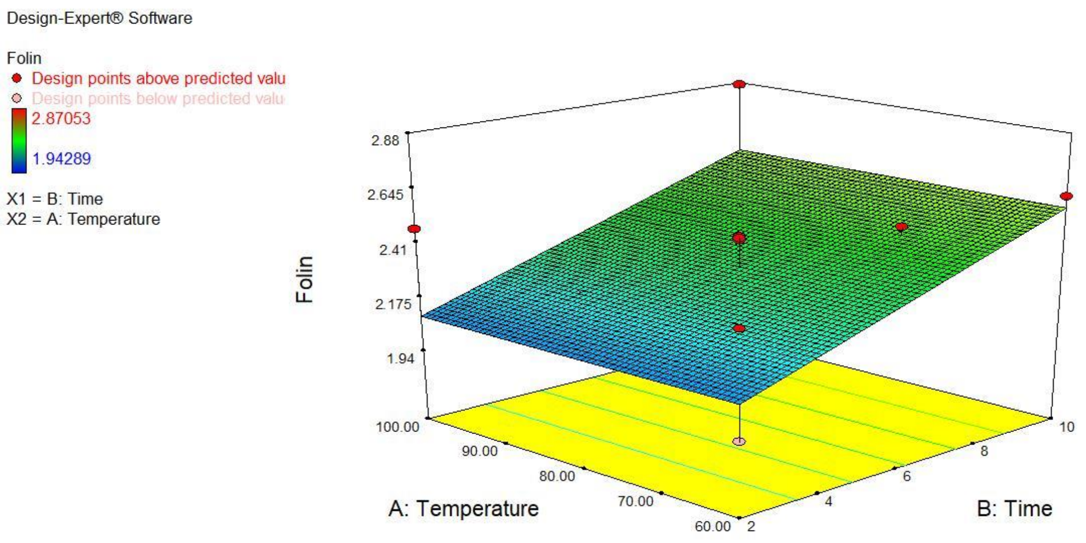

Figure S1. The response surfaces in the tests a) Folin-Ciocalteu b) ABTS. Parameter A – temperature, parameter B - time

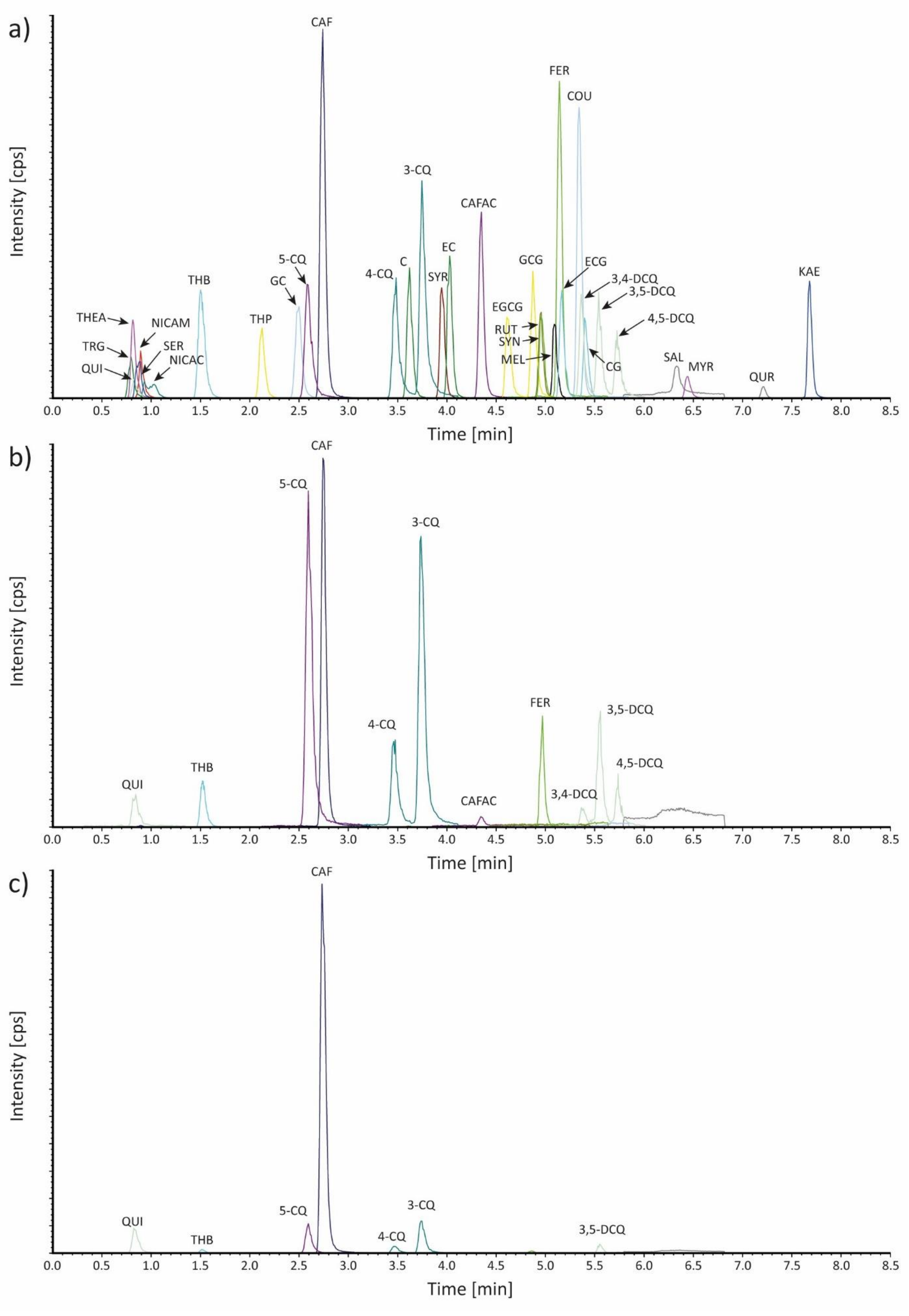

Figure S2. Chromatograms of a) standard solution, b) sample 10, c) sample 8
